# A Hybrid Optimization Algorithm for Water Volume Adjustment Problem in District Heating Systems

**DOI:** 10.1007/s44196-022-00091-8

**Published:** 2022-06-14

**Authors:** Yi Han, Pengfei Pan, Hexin Lv, Guoyong Dai

**Affiliations:** 1 Zhejiang Shuren University, Hangzhou, China; 2 Changzhou University, Changzhou, China

**Keywords:** Satisfaction, Polar bear optimization, Chemical reaction optimization, NSGAII, Multi-objective

## Abstract

Nowadays, the winter is getting harsher and harsher in Northern China. Thus, the centralized heating systems (CHSs) are playing even more irreplaceable, essential and critical roles in ensuring general public’s livelihood than never ever before. CHSs are normally composed of one or several combined heat and power (CHP) plants (units) and an extensive vein like district heating networks (DHNs) connecting with chemical plants, paper mills, food processing factories, hospitals, hotels, universities, prisons and residential complexes. A CHP plant in Northern China usually consumes coal to heat the cold water into steam to drive high-pressure turbines and low pressure turbines to generate electricity. Then the low-temperature steam is used to heat up the cold water in a main pipe into hot water travelling through the DHNs to provide heat to each end nodes. The returned water will be heated again for reuse and the surplus steam will be released into air through cooling towers. In 2020, China promised to the world that carbon dioxide will peak in 2030 and net-zero emission will happen in 2060. On the one hand, CHP plants need to guarantee enough hot water flowing within each household’s heating radiator. On the other hand, they should cut down on the consumption on non-renewable resources. Lowering water temperature, adjusting water volume and reducing water pressure will all contribute to energy-saving purpose. Lowering water temperature and reducing water pressure may cause too much heat losses during long-distance transmission in frigid winter. Therefore, a reasonable water volume adjustment becomes an advisable action comparatively. Here, we transfer the hot water supply volume optimization problem (HWSVOP) into a heat exchange station (HES) valve angle adjustment problem (SHWESVAAP). Then, a multi-objective mathematical model is established considering balancing the satisfactory degree of each household in residential quarters and the hot water volume (HWV) in the main pipe. And a hybrid polar bear optimization algorithm integrated with chemical reaction optimization (HA-PBO-CRO) is designed to optimize the valve angle (VA) in each HES. The comparative results between HA-PBO-CRO and non-dominant sorting genetic algorithm (NSGAII) demonstrate HA-PBO-CRO is superior to NSGAII with better Pareto frontiers on one hand and provide a critical reference supporting the management in a CHP plant to make a right decision on what to do to cut energy consumption while satisfying customers’ needs.

## Introduction

**Fig. 1:**
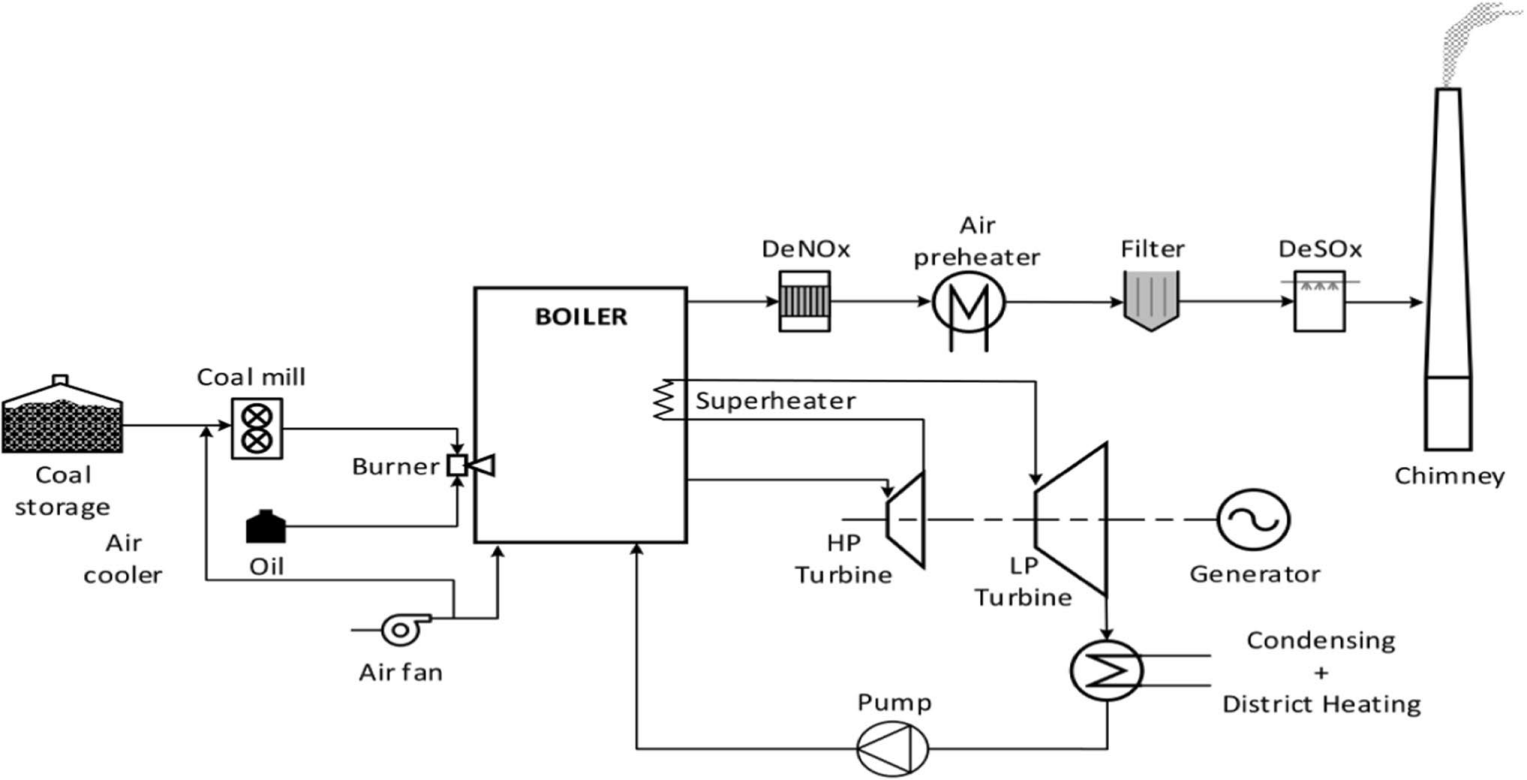
General operational mode-1 of a CHS [3]

**Fig. 2:**
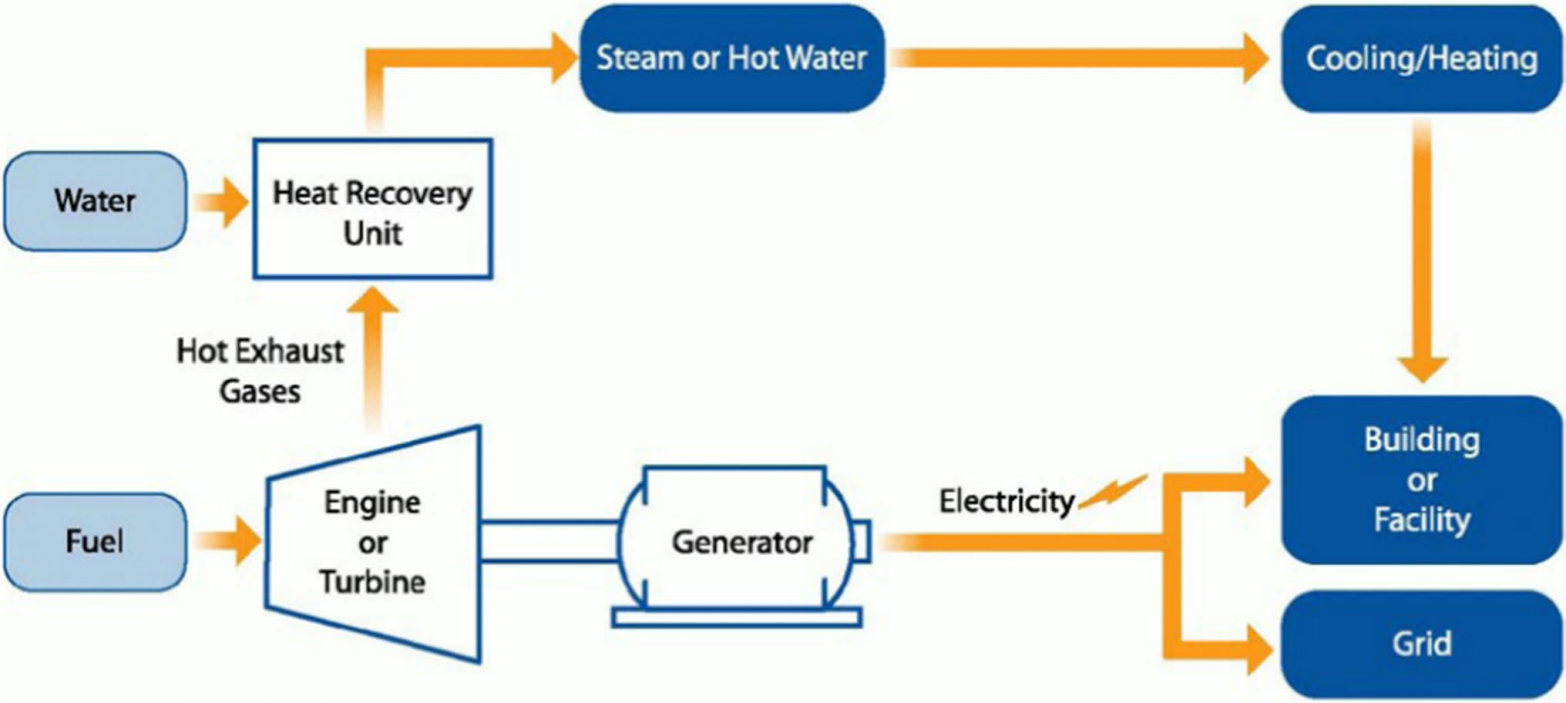
General operational mode-2 of a CHS [4]

Centralized heating system (CHS), which is composed of heat sources, heat exchange stations, heat users and district heating networks, is playing even crucial role in escorting northern people through unbearable and annoying cold weather [1]. In a CHS, there are high coupling relationships and great mutual influences among heat sources, networks and users. Normally, heat sources mean CHPs which operate in a combined heat and electricity generation mode producing hot water and electricity together by burning coals and other energy resources [2]. District heating networks mean the underground and built-in-buildings water pipes that reach to each radiator under the window of every northern family. Heat users mean those local residents who rely heavily on those CHPs to deliver hot water into their homes during winter time. The general operational modes are shown in Figs. 1, 2 [3, 4].

**Fig. 3:**
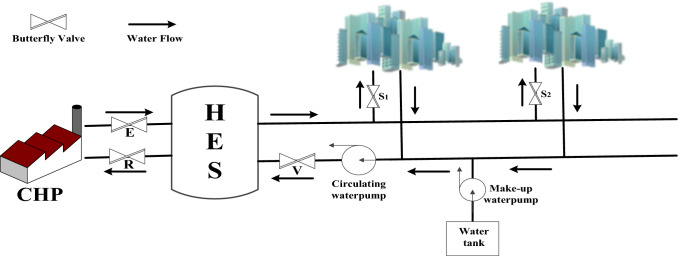
Process of heat exchange in a centralized heating system

The overheated hot water from a CHP plant which will go through an extraordinary wide main pipe cannot be directly pumped into each household. Traditionally, there are some HESs standing in between to neutralize the water temperature to an acceptable degree. And thereafter, the hot water in a HES is sent out to warm up each flat in a residential quarter. A simplified process showing how a HES works is shown in Fig. 3.

**Fig. 4:**
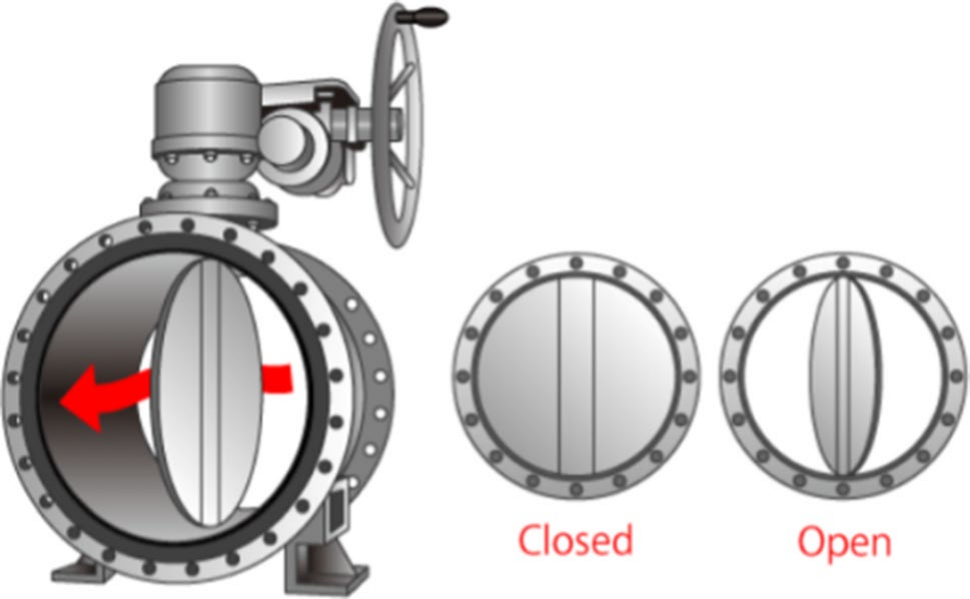
A Butterfly Valve

Right now, what are bothering those CHPs most in northern China is indeed how much hot water they should provide [5]. The reason is the more the hot water is, the more the electricity and energy consumption will be. With more and more greener energies like hydro, wind and solar powers enter into State GRID, more coalfire power means heavier penalties. In Fig. 3, the regular operations on regulating hot water volume are carried out by an experienced operator controlling the gate (disc) angle *Ɵ* of a butterfly valve (see Fig. 4). As a result, many complaints and grumbles from local people arrive in flocks since a manual adjustment fail to meet the majority’s satisfactions. In view of this, if we could set a wise gate (disc) angle *Ɵ* for valve *E*, we could help CHPs to make the right decision about how much hot water they need to deliver while pleasing the major customers and avoiding too much penalties from the government. Meanwhile, the energy consumption in CHPs will be further saved to comply with the appeal of China’s carbon peak and net-zero carbon discharge.

Here, a mathematical model, which is composed of minimizing users’ dissatisfactory degrees and hot water volume (HWV) as two objectives, was proposed after several years of field investigation in CR POWER group in Shenyang. Hundreds of residents’ satisfactory degree functions were obtained by collecting and clustering questionnaires from Dadong and Huanggu districts. HA-PBO-CRO was employed to optimize those valve gate angles, which have a direct effect on the users’ satisfactory degrees and HWV at each heat exchange station. Through comparing HA-PBO-CRO with NSGAII, it is easy to discover that HA-PBO-CRO is superior to NSGAII in effectiveness and efficiency. And the computational result is guidance for CHPs to reach sensible decisions.

## Literature Review

CHSs have a history of several hundred years. There are several components in CHSs. Hear source means CHPs that provide hot water in the main pipe. In Northern China, hot water is the main product and electricity is a byproduct that will infuse into state grid directly if no storage technologies were adopted. Today, the more the electricity from coal, the more the fine from Chinese government will be. Cutting hot water volume will be especially critical since it will indirectly reduce the fine for over supplying electricity. Heat network (HN) means a vast extensive underground water pipes going to each corner of several districts. HES is a node on the heat network linking the main heat network and the secondary network responsible for reducing the hot water temperature in the secondary heat network by adjusting heat exchanger’s valve angle to allow more or less hot water in the main pipe to flow in. The main heat network links the main pipes and the secondary network links the residential quarters or complexes. The radiators are end devices placed under each household’s windows to heat up the whole apartment.

Currently, some European countries like Russia, France and England are among the world’s leaders in utilizing CHS. In Russia, more than 70% residential buildings in 95% towns are based on CHS [6]. The Russian heating system has its own unique characteristics: high-quality heating equipment, mature technology for the operation and adjustment, and a high degree of flexibility and automation. Similarly, in Denmark, 70% of the population is benefiting from CHS, where one CHP plant could show off a productivity of up to 90%.

CHS in China has a history of 80 years from 1941. The booming period is after the founding of New China, when economic development continued to develop and the construction of infrastructure was in its full pace. Beijing, Shenyang, Taiyuan and other places with the geographical advantage of being close to the coal mines have built a variety of thermal power plants with different scales. These CHP plants contributed a great deal to supply heat to surrounding neighborhoods. According to statistics, CHS accounts for 62.9 percent of the total heat capacity in China [7].

In CHS related researches, scholars mainly focused their attentions on supplying heat on demand and volume adjustment aspects. The mathematical models were single or multiple objectives and the solving tools were mostly meta-heuristics or intelligent optimization algorithms.

Supplying heat on demand has always been a research focus of scholars all over the globe. Woznick et al. [8] applied polar bear algorithm to analyze and calculate the effect of outdoor temperature changes in Germany and Finland. They proposed to use the circulating pump in the heat exchange station to implement on-demand heat supply. Similarly, Turski et al. [9] adopted heat load prediction method to achieve dynamic adjustment in heating stations according to climatic conditions and heat users’ demands. Zhong et al. [10] put forward the concept of "smart heating" under the background of “Internet + ”. They suggested that with big data, artificial intelligence, simulation and other technologies, CHS can make corresponding self-adjustment and self-adaptation according to actual needs. They applied their theory to a CHS established in Beijing hiring online simulation means and intelligent algorithms to operate real-time multi-source heat load dispatching.

Volume adjustment is a key factor in the operation in CHS. Zhao et al. [11] proposed to install regulating valves in the steam heating network. And they calculated the ideal valves’ opening angles with parallel genetic algorithm to change the distribution of steam flow in an industrial park in Shanghai. Coal consumption is an important index to measure the cost of CHS. Wang et al. [12] applied particle swarm optimization algorithm to power load distribution with the optimization goal of minimizing the total coal consumption. They proved their theory by comparing the total daily coal consumption data from before and after optimization. Ikonen et al. [13] established a dynamic linear model minimizing the operating cost of regional heating supply system and verified the effectiveness of their model by simulation computation.

As time went by, the focus shifted from single objective problems to multiple objective ones. Ju [14] established a multi-objective stochastic dispatching model minimizing the operating cost and load fluctuation in virtual thermal power plants and proved the effectiveness and practicability of their method. Wei [15] used a multi-objective interval optimization method to solve the synchronized energy capacity allocation optimization problem in CHS and evaluated Pareto optimal solution set from the perspective of cost, risk and emission with dynamic game theory. Zhang et al. [16] put forward a method combining multi-objective optimization with integrated decision-making to solve economical discharge problem considering different heat demands under changing conditions. Chen et al. [17] established a bi-objective model to maximizing economic benefit and minimizing environmental cost in CHS. An improved particle swarm optimization algorithm was adopted to optimize heat load distribution. The overall benefit of the optimized load distribution plan was better than workers' routine operation. He [18] established a stochastic model with the objective function minimizing the total cost of CHS and the total amount of exhaust emissions.

Meta-heuristics are random search methods mimicking some animal behaviors or physical and chemical phenomena. Although Meta-heuristics may not guarantee to find the optimal solutions, they still possess unique and irreplaceable advantages. For example, they could solve problems without requiring the objective functions and constraints to be continuous and convex.

Since Genetic algorithm was proposed in 1975 [19], many novel meta-heuristics and their hybrid versions emerged in abundance. In 1983, Kirkpatrick et al.[20] invented a simulated annealing (SA) algorithm from the annealing process of metals. In 1991, Maniezzo [21] put forward an ant colony optimization (ACO) algorithm according to the phenomenon of ants foraging process. In 1995, Eberhart and Kennedy [22] devised particle swarm optimization (PSO) following the social behavior of birds. In 2007, Karaboga et al. [22] created artificial bee colony algorithm (ABC) based on honey collection activities. In 2009, Yang [23] coded firefly algorithm (FA) based on the social behavior of fireflies. In 2010, Albert Lam and Victor Li [24] publicized a chemical reaction optimization algorithm (CRO) in view of the intermolecular interaction in chemical reactions. In 2012, Pan [25] described a Fruit Fly Optimization Algorithm (FFOA) considering the foraging behavior of fruit flies. In 2013, Kaveh and Farhoudi [26] exhibited a dolphin echolocation algorithm (DE) according to the dolphin’s ability to locate food in the sea. In 2015, Mirjalili [27] developed a dragonfly algorithm (DA) by observing the static and dynamic clustering behaviors of dragonflies. In 2017, a butterfly algorithm appeared from the butterfly mating mechanism [28]. David and Marchi [29] produced a polar bear optimization (PBO) in 2017. Zeng et al. [30] showed a whale swarm algorithm in 2017. Gravitational search algorithm came into being in 2009 based on the Newtonian gravity and the laws of motion [31]. An antlion optimizer (ALO) was discovered in 2015 displaying hunting mechanism of antlions in nature [32]. Harris Hawks Optimization (HHO), which was introduced by Heidari et al. in 2019, featured the cooperative behaviors and chasing style of hawks [33]. Teaching–Learning (TL) algorithm gained attentions from scholars in 2011 carving out the process of teachers’ teaching effects and students’ learning efficiency [34].

As is known to all, there is a ‘no-free-lunch’ theory claiming no algorithm is suitable for all optimization problems. After single versions were known to the world, many hybrid algorithms came into being afterwards to improve the performances of single versions. Garg [35] hybrid PSO and GA together for a series of constrained optimization problems in 2016. Then, Garg [36] integerated GA with GSA to solve constrained optimization problems in 2019. In 2021, Kundu and Garg [37] improved TL algorithm and bound it together with HHO for numerical and engineering optimization problems.

All of the abovementioned hybrid algorithms achieved better results. Therefore, we adopted a hybrid algorithm to solve VAAP.

## Problem Descriptions and Mathematical Model

Through an extensive investigation on a cogeneration power plant in Shenyang, we found that the main problems in power plants are low customer satisfaction and excessive power supply to the State Grid, which leads to fines of several millions to dozens of millions of RMB.

In the centralized heating system, HESs connect with the primary pipe and the secondary heat network. HESs can have an indirect effect on the water supply temperature and the backwater temperature in the secondary network and the users’ indoor temperature according to the different weather conditions and changes of users' demands by adjusting the volume of hot water entering HESs. Adjusting hot water volume means changing the valve disc angle $\theta$ of each HES. Normally, during the valve disc angle adjustment process in a HES, the operators turn the wheel on the valve to increase or reduce the entering water volume according to their own experiences. This empirical behavior could not satisfy the needs of heat users very well. Thus, the complaints are growing and the satisfaction degrees of heat users keep dropping down in recent years.

**Fig. 5:**
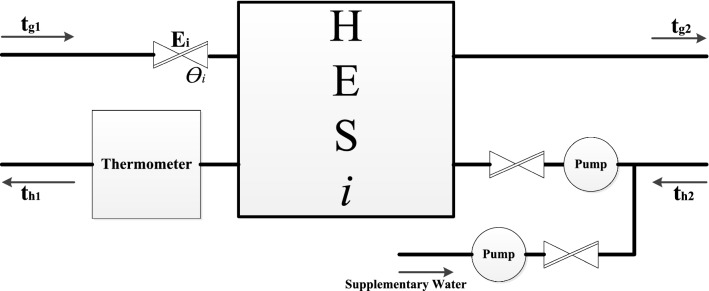
A simplified HES system

A HES system is simplified in Fig. 5. Here, *E*_*i*_ is the entering water valve of HES_i_; $\theta_{{\text{i}}}$ is the opening angle of *E*_*i*_; *t*_*g*1_ is the entering water temperature in the primary network; t_g2_ is the entering water temperature in the secondary network; *t*_*h*1_ is the backwater temperature in the primary network, and *t*_*h*2_ is the backwater temperature in the secondary network.

Assume that the heat loss is zero throughout the centralized heating system. Then, the heat *Q* in the pipe could be calculated with formula (1) [38–42]. Here, *V* is circulating water volume; *C* is the specific heat capacity; *t*_*g*_ is entering water temperature and *t*_*h*_ is backwater temperature.

1 $$ {\text{Q}} = {\text{G}} \cdot {\text{C}} \cdot \left( {t_{g} - t_{h} } \right) $$

From the view of heat balance, if the heat loss of hot water in the pipe is neglected, formula (2) will stand.

2 $$ {\text{C}} \cdot V_{1} \cdot t_{g1} - t_{h1} = {\text{C}} \cdot V_{2} \cdot \left( {t_{g2} - t_{h2} } \right) $$

Here, *V*_1_ is the water volume entering into HES from the primary network and *V*_2_ is the water volume entering into HES from the secondary network. And, the relationship between *t*_*g*2_ and *V*_1_ can be obtained as follows: $t_{g2} = \frac{{\left( {t_{g2} - t_{h2} } \right)}}{{V_{2} }} \cdot V_{1} + t_{h1}$. Suppose *V*_2_, *t*_*g*2_, *t*_*h*2_ and *t*_*h*1_ are constants. Then, $t_{g2} = a \cdot V_{1} + {\text{b}}$. Therefore, adjusting *V*_1_ means controlling *t*_*g*2_.

When the heating system is in a stable state, there is a balanced relationship between the indoor temperature *T*_*n*_ gained from the radiator at the user’s end and *t*_*g*2_ (see formula (3)) [40–42].

3 $$ {\text{ T}}_{{\text{n}}} { = }\frac{{{\text{KF}}_{r} }}{{KF_{r} + KF_{b} }} \times \frac{1}{2}(t_{g2} + t_{h2} ){ + }\frac{{{\text{KF}}_{b} }}{{KF_{r} + KF_{b} }} \times T_{w} \, $$

Here, *KF*_*r*_ is the heat dispersing coefficient of a radiator; *KF*_*b*_ is the heat dispersing coefficient of a building and *T*_*w*_ is the outdoor temperature. Suppose *KF*_*r*_, *KF*_*b*_, *t*_*h*2_ and *T*_*w*_ are constant. Then, the relationship between *T*_*n*_ and *t*_*g*2_ is like ${\text{T}}_{n} = c \cdot t_{g2} + {\text{d}}$. Furthermore, we can get ${\text{T}}_{n} = c \cdot a \cdot {\text{V}}_{1} + c \cdot b + {\text{d}} = {\text{e}} \cdot {\text{V}}_{1} + z$. Therefore, changing *V*_1_ means adjusting *T*_*n*_.

In this paper, we consider how to optimize the water supply volume while satisfying the users’ satisfaction with the indoor temperatures. We establish fuzzy trapezoidal functions with ten types according to the users’ preferences summarized in the questionnaire. By adjusting the water supply volume from the primary network into the HES, the indoor temperature at a user’s house can be indirectly affected and the satisfaction degree of this user could be obtained as well. We assume that the opening angle of each valve in the HES systems is ${90}^{^\circ }$, and only the opening of the entering water valve disc angle of each HES needs to be optimized. In addition, we assume that the indoor temperature is also affected by the distance, namely ${\text{T}}_{{{\text{nr}}_{ij} }} = {\text{T}}_{{{\text{n}}_{ij} }} - \frac{{{\text{D}}_{ij} }}{100}$. Here *D*_*ij*_ (meters) is the distance between a user *j* and a HES *i*.

A bi-objective optimization model is adopted to represent the real operational problem and $x_{i}$ is used as the optimization variable $\left( {x_{i} = \frac{{\theta_{i} }}{{\theta_{max} }} = \frac{{V_{{1_{i} }} }}{{V_{max} }} = \frac{{\theta_{i} }}{{90^{^\circ } }}} \right)$. Here, $x_{i}$ represents the ratio of the opening angle $\theta_{i}$ of the inlet valve at the *i*th HES to 90 degrees. The smaller the $x_{i}$, the lower the water supply volume and the indoor temperature. Then, ${\text{T}}_{{{\text{nr}}_{ij} }} = e \cdot x_{i} \cdot V_{max} + {\text{z}} - \frac{{{\text{D}}_{ij} }}{100}$. Formula (4) indicates that the average value of the users’ dissatisfaction degrees should be as small as possible. And Formula (5) exclaims that the average number of total water supply volumes of all HESs should be as small as possible.

4 $$ f_{1} = 1 - {\text{max}}\{ \frac{1}{MN}\mathop \sum \limits_{i}^{N} \mathop \sum \limits_{j}^{M} FF_{ijk} \left( {e \cdot x_{i} \cdot V_{max} + {\text{z}} - \frac{{{\text{D}}_{ij} }}{100}} \right)\} $$

5 $$ f_{2} = \min \{ \frac{1}{N}\mathop \sum \limits_{i}^{N} x_{i} \} , $$

where *N* is the total number of HESs; *M* represents the total number of users and *FF*_*ijk*_ is the *k-*th type satisfaction function value corresponding to the indoor temperature at the *j*-th user in the *i*-th HES secondary network system.

## Multi-Objective Optimization and NSGA-II

In real life, when several objectives are optimized, conflicts will happen. When the solution of one objective is improved, it may harm the other objectives. Multi-objective optimization is an optimization process of finding solutions optimizing all objectives as much as possible. The solution set of multi-objective optimization problem is called Pareto optimal solution set, which was first put forward by a Italian economist Pareto in 1906 [43]. Finding a set of solutions is the ultimate goal of Pareto optimal process. The multi-objective optimization problem (MOP) is described as follows: There are *p* inequality constraints and *q* equality constraints.

6 $$ {\text{min}}\,f\left( x \right) = \left[ {f_{1} \left( x \right),f_{2} \left( x \right), \cdots ,f_{m} \left( x \right)} \right] $$

7 $$ {\text{s}}.{\text{t}}.{ }\,h_{i} \left( x \right) \le 0,i = 1,2, \cdots ,p $$

8 $$ g_{i} \left( x \right) = 0,i = 1,2, \cdots ,q $$

### Multi-Objective Important Concepts and Definitions [43]

Definition 1(Pareto domination x≺y):

Let *x* and *y* be two different solutions in a population. If f_*i*_(*x*) ≤ f_*i*_(*y*) for all objectives, *x* is called the non-dominant solution and *y* is the dominant solution.

Definition 2 (Non-dominant solution set):

In a solution population, the set consisting of solutions which could not be dominated by any other solutions is called a non-dominant solution set.

Definition 3 (feasible solution):

When a solution in the decision space meets all constraints, this solution is called a feasible solution.

Definition 4 (Pareto optimal solution):

Let *x* be a feasible solution. When there are no solutions that can dominate *x*, *x* is a Pareto optimal solution.

Definition 5 (Pareto optimal solution set):

A set of all Pareto optimal solutions is a Pareto optimal solution set.

**Fig. 6:**
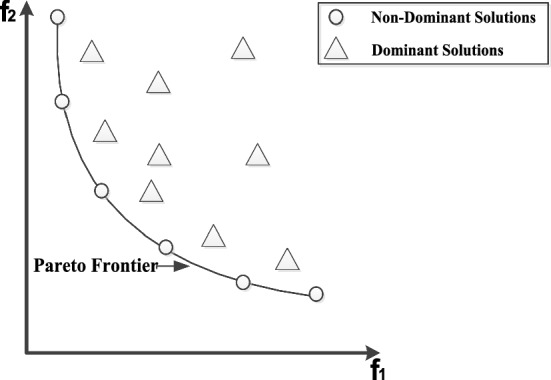
Pareto frontier and dominant solutions

Definition 6 (Pareto frontier):

The objective values of all solutions in Pareto solution set in a coordinate space constitute a Pareto frontier. Figure 6 clearly describes the relationship between the dominant solutions and the Pareto frontier for a bi-objective optimization problem.

### NSGA-II

With the continuous development of multi-objective research, many multi-objective optimization algorithms emerge. NSGA-II is one of those multi-objective optimization algorithms with ideal results. Thus, it is often used as a benchmark for comparing the calculation results of other algorithms. NSGA-II means non-dominated sorting genetic algorithm with elite mechanism. It was put forward by Deb et al. [44] based on NSGA in 2000. NSGA-II overcomes three shortcomings of NSGA: (1) optimal solutions retention mechanism; (2) adaptive setting for sharing parameters; (3) computation complexity [45–48]. In NAGA-II, the evolutionary population is divided into several layers by dominance relations. The first layer is the set of non-dominant individuals and the second layer is the set of non-dominant individuals obtained by removing those individuals from the first layer [46]. The advantages of NAGA-II algorithm are as follows: (1) Introducing the elite retention mechanism to expand the population space, and combining the parent and child generations to compete together to produce the next generation population preventing the optimal solution from being missed out; (2) The non-dominated sorting speed is improved by reducing the computational complexity from O(*n*^3^) to O(*n*^2^); (3) Crowding degree can keep the distribution and diversity of solution population and can avoid sharing parameter selection [43].

**Fig. 7:**
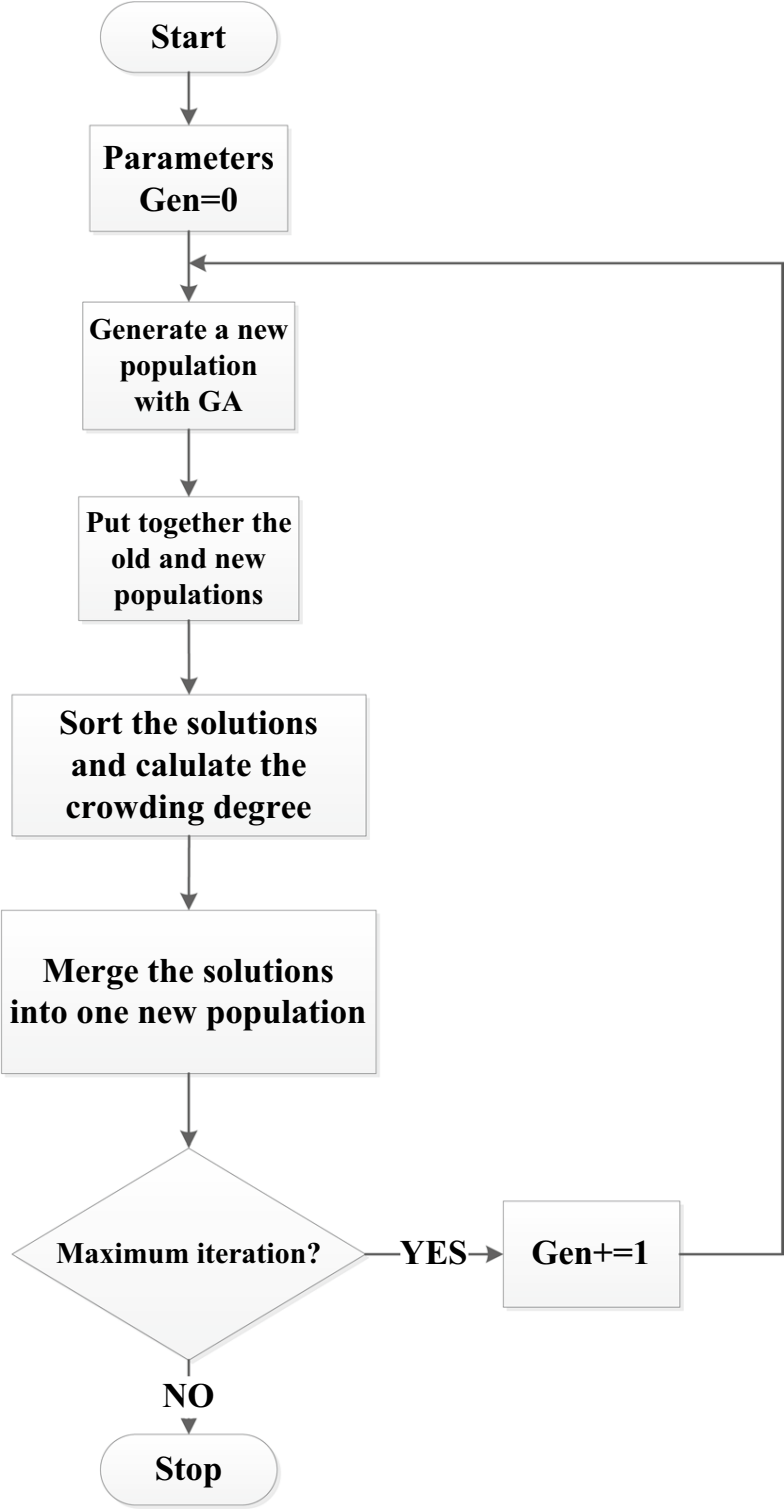
Flowchart of NSGA-II

The flowchart of NSGA-II is as shown in Fig. 7.

## Polar Bear Optimization and Chemical Reaction Optimization [29]

### Polar Bear Optimization

**Fig. 8:**
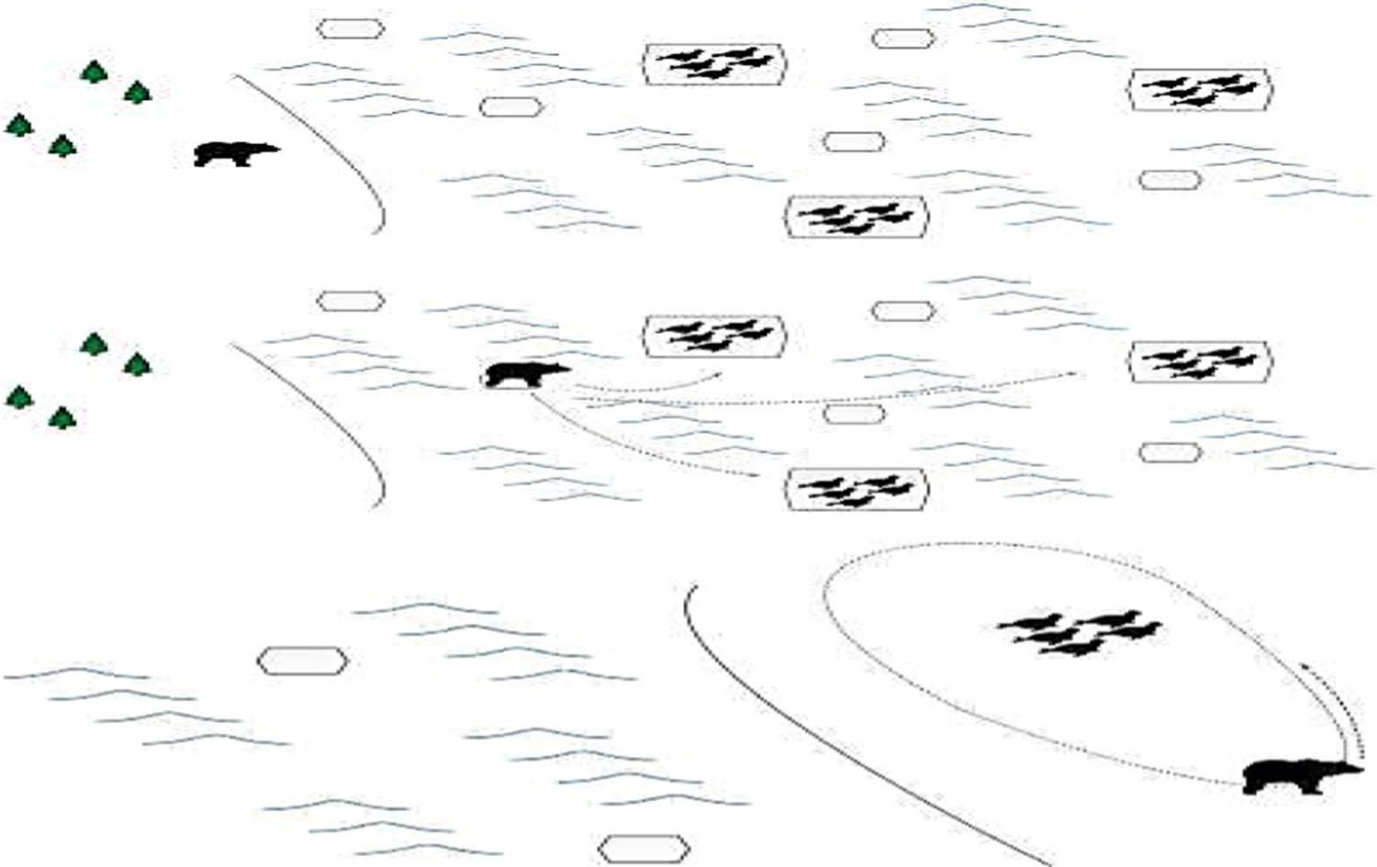
Schematic diagram of polar bear seeking and predating on seals [29]

Polar Bear Optimization (PBO), which is one of the bio-inspired meta-heuristic algorithms, was proposed by David et al. in 2017 [29]. The idea of the algorithm came from the foraging process of polar bears (see Fig. 8).

The core of polar bear algorithm is composed of ice floe floating strategy, seal predation strategy and bear colony breeding and death strategy.

Ice floe floating strategy is a global search mechanism which can be represented by formula (9). Here, *β* is a random number in the interval (0, 1) and *γ* is a random number in the interval [0,|ω|]. *sign*(ω) is a piecewise function with a result {–1, 0, 1} changing with *ω* {negative, 0, positive}. *ω*, which is depicted in formula (10), is the Euclidean distance between two polar bears.

9 $$ (x_{j}^{i} )^{t} = (x_{j}^{i} )^{t - 1} + sign(\omega ) \cdot \beta + \gamma $$

10 $$ \omega = \sqrt {\sum\limits_{k = 0}^{n - 1} {(x_{k}^{best} - x_{k}^{i} )^{2} } } $$

Seal predation strategy serves as local search mechanism. The hunting mode of each polar bear can be expressed by formula (11). In the searching process, the radius of view is denoted by the following two parameters: $a \in \left[ {0,0.3} \right]$ limits the visible distance; $\emptyset_{0} \in \left( {0,\frac{{\uppi }}{2}} \right)$ indicates the oblique angle at which a polar bear travels around prey.

11 $$ r = 4a\cos \left( {\emptyset_{0} } \right)\sin \left( {\emptyset_{0} } \right) $$

During the movement of each polar bear, the updating process of coordinates in different dimensions is shown in formula (12). Here, $\emptyset_{1} ,\,\emptyset_{2} , \ldots ,\emptyset_{n} \in \left( {0,\,2{\uppi }} \right)$.

12 $$\left\{\begin{array}{c}{x}_{0}^{new}={x}_{0}^{old}\pm r\mathrm{cos}({\varnothing }_{1})\\ {x}_{1}^{new}={x}_{1}^{old}\pm r[\mathrm{sin}({\varnothing }_{1})+\mathrm{cos}({\varnothing }_{2})]\\ {x}_{2}^{new}={x}_{2}^{old}\pm r[\mathrm{sin}({\varnothing }_{1})+\mathrm{sin}({\varnothing }_{2})+\mathrm{cos}({\varnothing }_{3})]\\ \cdots \\ {x}_{n-2}^{new}={x}_{n-2}^{old}\pm r[\sum_{k=1}^{n-2}\mathrm{sin}({\varnothing }_{k})+\mathrm{cos}({\varnothing }_{n-1})]\\ {x}_{n-1}^{new}={x}_{n-1}^{old}\pm r[\sum_{k=1}^{n-1}\mathrm{sin}({\varnothing }_{k})+\mathrm{cos}({\varnothing }_{n})]\end{array}\right.$$

If the updated position of a polar bear is better than the current position, “ + ” sign is applied to indicate a forward move; if the updated position is not ideal, a polar bear will go in the direction of “−”. When the results in both directions are not acceptable, the polar bear stays where it is.

Bear colony breeding and death strategy, which is a population updating mechanism, is indicated by formula (13), (14).

13 $$ \left\{ {\begin{array}{*{20}c} {Death\,if\,k < 0.25} \\ { Reproduction\,if\,k > 0.75} \\ \end{array} } \right. $$

14 $$ \left( {x_{j}^{reproduction} } \right)^{t} = \frac{{\left( {x_{j}^{best} } \right)^{t} + \left( {x_{j}^{i} } \right)^{t} }}{2} $$

In formula (13), *k* is a random number in the interval [0, 1]. If the death mechanism is triggered, a weakest polar bear will die if the population number is more than 50% of the maximum population capacity *n*. In formula (14), in the *t*-th iteration, one of top 10% individuals in the population will reproduce and the population will expand if the population size is less than *n*-1.

The executive steps of PBO are as follows:

1. Setting parameters;
2. Randomly generate 75% of population capacity *n* and record the best solution;
3. For each polar bear, randomly set the angle of each dimension;
4. Use formula (11) to calculate the search radius *r*;
5. Use “ + ”to calculate the new position of a polar bear by formula (12) and update the current position if the new position is better than the current position;
6. Calculate the new position of a polar bear by formula (12) in the direction of “−” and update the current position if the new position is better;
7. According to formula (9) and ω, determine the new position of each polar bear;
8. Sort the population and select the first polar bear to judge whether to update the global best solution;
9. Randomly select a solution different from the global best solution from top 10% in the current population;
10. Randomly generate *k*. if *k* < 0.25 and the population size is greater than 50% of the population capacity n, remove the solution that ranked last;
11. If the population size is less than *n*-1, generate a new bear to join the population according to formula (14);
12. Judge whether the maximum iteration number has been reached. if yes, go to step.13, if no, go to step.3;
13. OuCHPut the global best solution and fitness value.

### Chemical Reaction Optimization [24]

Chemical reaction optimization (CRO) was designed in 2010 [24]. CRO mimics the process that molecules react in a closed container and finally reach a low-energy stable state. In CRO, a molecular structure ($\varphi$) represents a candidate solution. The potential energy (PE) of a molecule represents the objective function value $f$ (see formula (15)). Kinetic energy (KE) determines the vitality of molecules and represents the tolerance of accepting a molecule worse than the current one. The greater the KE is, the stronger the ability to escape from local minimum will be. The molecular elements are composed of φ, PE, KE, Number of total hits (number of hits), current best solution (minimum structure), current best objective function value (minimum value) and number of hits when best solution is achieved (minimum hit number). In a chemical reaction, kinetic energy and potential energy can be mutually transformed into each other within or between molecules. According to the energy conservation law, if chemical reactions take place in a closed system, energy can only be transferred from one form to another or from one molecule to another. And the total energy will not change. Therefore, in CRO algorithm an energy buffer (EB) is used to store part of the kinetic energy in a molecule. A molecule $\varphi$ is allowed to change into $\varphi ^{\prime}$ if ${\text{PE}}_{\varphi } \ge {\text{PE}}_{\varphi ^{\prime}}$ or ${\text{PE}}_{\varphi } + {\text{KE}}_{\varphi } \ge {\text{PE}}_{\varphi ^{\prime}}$.

15 $$ {\text{PE}}_{\varphi } = f\left( \varphi \right) $$

**Fig. 9:**
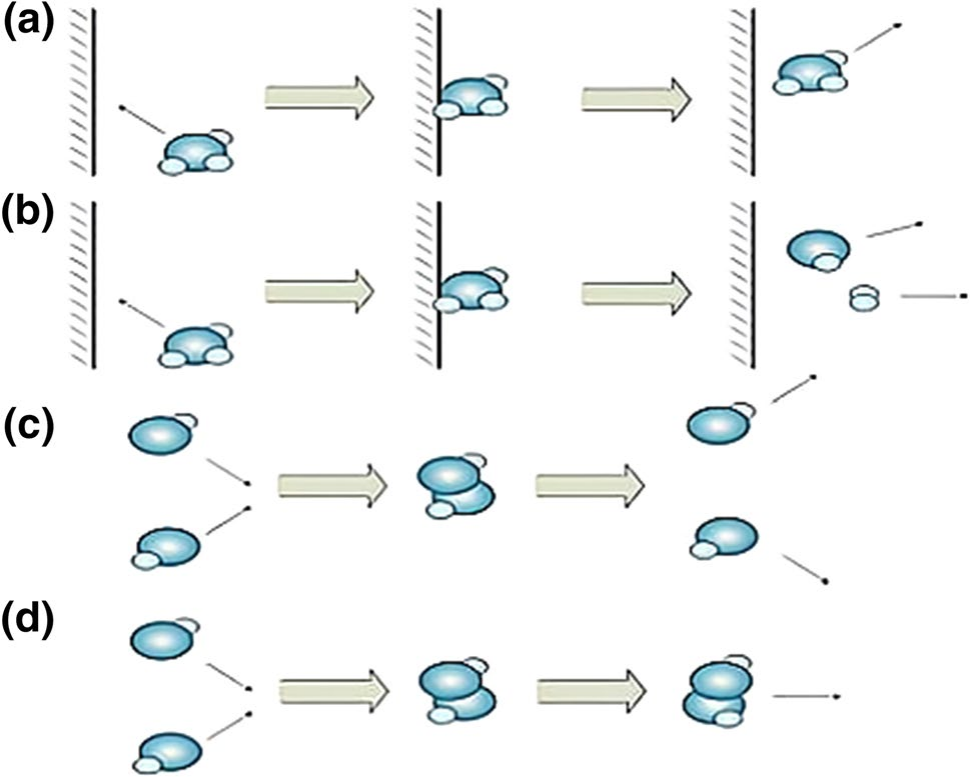
Four basic reactions in CRO [24]

There are four basic reactions in CRO as follows: (a) On-wall ineffective collision; (b) Decomposition; (c) Inter-molecular ineffective collision; (d) Synthesis (see Fig. 9).

In on-wall ineffective collision, Molecules bounce back after hitting the container wall. During collision, the structure of a molecule may remain unchanged or be changed. The molecular structure $\varphi$ changes into $\varphi ^{\prime}$ after the collision, if the relationship between energies satisfies formula (16). The new ${\text{KE}}_{\varphi ^{\prime}}$ can be obtained with $KE_{\varphi ^{\prime}} = \left( {PE_{\varphi } + KE_{\varphi } - PE_{\varphi ^{\prime}} } \right) \times q$, where *q*$\in$[KElossrate,1] and (1-q) represents the loss of KE when collision happens. KElossrate is a parameter whose value satisfies 0 < KElossrate < 1. The lost KE will be stored in the central EB, which will support decomposition. The more the collision happens, the more KE is stored in the central EB. If formula (16) is not met, a molecule will keep its original structure, PE and KE.

16 $$ {\text{PE}}_{\varphi } + {\text{KE}}_{\varphi } \ge {\text{PE}}_{\varphi ^{\prime}} $$

Decomposition starts when a molecule hits the container wall and decomposes into two independent molecules. In CRO, decomposition reaction needs to satisfy formula (17). The KEs of two new molecules are calculated using formula (18), (19) with k$\in$[0, 1]. If formula (16) is not met, the KE stored in the central EB can be used to support the decomposition reaction according to formula (20). The KEs of two new molecules are then calculated by formula (21), (22) with (m_1_, m_2_, m_3_, m_4_)$\in$[0,1]. If formula (20) is not true, the decomposition reaction will fail to happen.

17 $$ PE_{\varphi } + KE_{\varphi } \ge PE_{{\varphi_{1}^{^{\prime}} }} + PE_{{\varphi_{2}^{^{\prime}} }} $$

18 $$ KE_{{\varphi_{1}^{^{\prime}} }} = \left( {PE_{\varphi } + KE_{\varphi } - PE_{{\varphi_{1}^{^{\prime}} }} - PE_{{\varphi_{2}^{^{\prime}} }} } \right) \times k $$

19 $$ KE_{{\varphi_{2}^{^{\prime}} }} = \left( {PE_{\varphi } + KE_{\varphi } - PE_{{\varphi_{1}^{^{\prime}} }} - PE_{{\varphi_{2}^{^{\prime}} }} } \right) \times \left( {1 - k} \right) $$

20 $$ PE_{\varphi } + KE_{\varphi } + buffer \ge PE_{{\varphi_{1}^{^{\prime}} }} + PE_{{\varphi_{2}^{^{\prime}} }} $$

21 $$ KE_{{\varphi_{1}^{^{\prime}} }} = \left( {PE_{\varphi } + KE_{\varphi } + buffer - PE_{{\varphi_{1}^{^{\prime}} }} + PE_{{\varphi_{2}^{^{\prime}} }} } \right) \times m_{1} \times m_{2} $$

22 $$ KE_{{\varphi_{2}^{^{\prime}} }} = \left( {PE_{\varphi } + KE_{\varphi } + buffer - PE_{{\varphi_{1}^{^{\prime}} }} + PE_{{\varphi_{2}^{^{\prime}} }} } \right) \times m_{3} \times m_{4} $$

The inter-molecular ineffective collision occurs when two molecules collide with each other and then bounce off. The KEs in this reaction will not go into central EB. Assume that the original molecular structures are $\varphi_{1}$ and $\varphi_{2}$. During the collision, two new molecular structures $\varphi_{1}^{^{\prime}}$ and $\varphi_{2}^{^{\prime}}$ are obtained from the neighborhood when formula (23) is satisfied. Two KEs of new molecules are calculated using formula (24) and formula (25) with *p*$\in$[0, 1].

23 $$ PE_{{\omega_{1} }} + PE_{{\omega_{2} }} + KE_{{\omega_{1} }} + KE_{{\omega_{2} }} \ge PE_{{\omega_{1}^{^{\prime}} }} + PE_{{\omega_{2}^{^{\prime}} }} $$

24 $$ KE_{{\omega_{1}^{^{\prime}} }} = (PE_{{\omega_{1} }} + PE_{{\omega_{2} }} + KE_{{\omega_{1} }} + KE_{{\omega_{2} }} - PE_{{\omega_{1}^{^{\prime}} }} + PE_{{\omega_{2}^{^{\prime}} }} ) \times p $$

25 $$ KE_{{\omega_{2}^{^{\prime}} }} = (PE_{{\omega_{1} }} + PE_{{\omega_{2} }} + KE_{{\omega_{1} }} + KE_{{\omega_{2} }} - PE_{{\omega_{1}^{^{\prime}} }} + PE_{{\omega_{2}^{^{\prime}} }} ) \times \left( {1 - p } \right) $$

Synthesis reaction forms when two molecules collide and merge into one molecule. Assume that the molecular structures of two molecules are $\varphi_{1}$ and $\varphi_{2}$_,_ respectively. During the collision, the existing two molecular structures synthesized into one molecular structure $\varphi ^{\prime}$ when formula (26) is satisfied. And the KE of the new molecule is calculated using formula (27).

26 $$ PE_{{\omega_{1} }} + PE_{{\omega_{2} }} + KE_{{\omega_{1} }} + KE_{{\omega_{2} }} \ge PE_{{\omega^{\prime}}} $$

27 $$ KE_{{\omega^{\prime}}} = PE_{{\omega_{1} }} + PE_{{\omega_{2} }} + KE_{{\omega_{1} }} + KE_{{\omega_{2} }} - PE_{{\omega^{\prime}}} $$

**Fig. 10:**
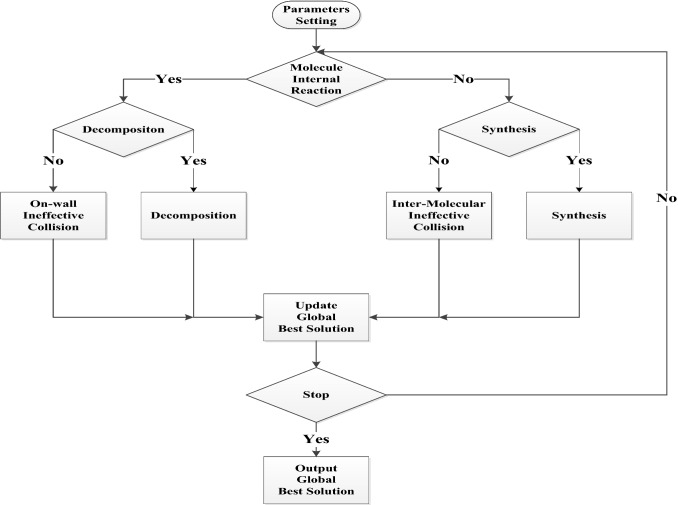
Flowchart of CRO

Looking through all four reactions, they are just criteria judging if new solutions should derive from old ones. However, there are no exact rules about how a solution should be changed into a new one. Therefore, CRO serves as a flexible framework accepting any possible detailed solution transformation operations. The simplified flowchart of CRO is shown in Fig. 10.

## A Hybrid Algorithm of Polar Bear Optimization and Chemical Reaction Optimization

In this paper, the adjustment decision of HWV considering customer satisfaction is considered and a hybrid algorithm of polar bear optimization chemical reaction optimization (HA-PBO-CRO) is applied to optimize a multi-objective optimization model.

### Solution Representation

In HA-PBO-CRO, a solution is coded as S = (x_1_, x_2_, x_3_,…, x_n_) with x_i_$\in$[0, 1]. If *x*_*i*_ = 0.3, the opening angle of *i*-th HES is 0.3*$90^{^\circ }$=$27^{^\circ }$.

### Modified On-Wall Ineffective Reaction

In this part, ice floe floating strategy in PBO is adopted to generate a new solution.

### Modified Decomposition

Here, seals predatory strategy in PBO is applied to produce two other solutions.

### Modified Inter-Molecular Ineffective Collision

In GA, crossover operation is often performed to turn two solutions into two different ones. Therefore, a crossover operator should fit well with this type of collision reaction.

### Modified Synthesis

Suppose two solutions are like *S*_1_ = (0.3, 0.5, 0.2, 0.6, 0.1) and *S*_2_ = (0.4, 0.5, 0.9, 0.7, 0.8). By adding *S*_1_ and *S*_2_ together, we get *S*_0_ = (0.7, 1, 1.1, 1.3, 0.9). However, there are two numbers that are greater than 1. Therefore, those two numbers are subtracted by 1. And *S*_0_ is set as (0.7, 1, 0.1, 0.3, 0.9).

**Fig. 11:**
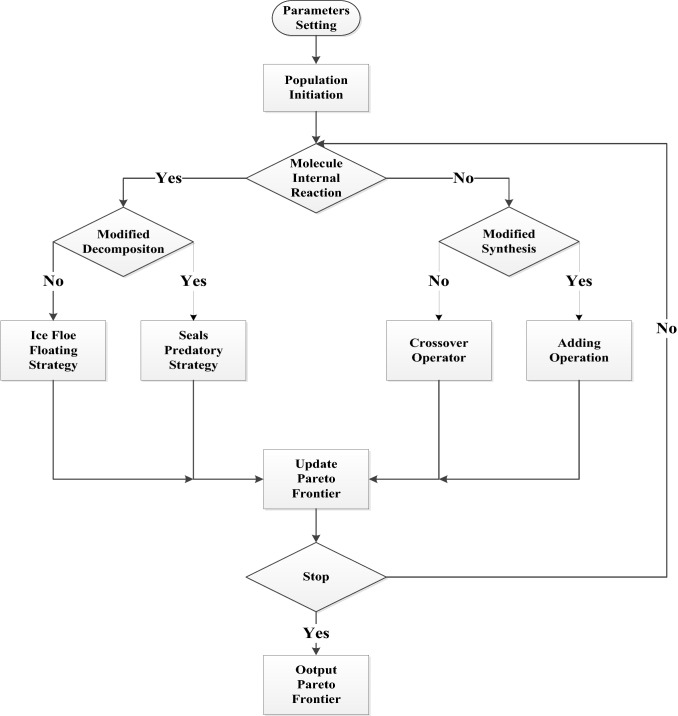
Flowchart of HA-PBO-CRO

The flowchart of HA-PBO-CRO is shown in Fig. 11.

## Simulation

Here, Shenyang China Resources Thermal Power Plant (SCRCHP) at Dadong district in Shenyang is selected as a case. SCRCHP has three 200 MW heat units installed with six large surface heat exchangers with heat transfer areas of 2600 m^2^. And the current heat network covers a radius of 15 km^2^. Shenyang is an area with an average annual temperature of 8.4 °C and four distinct seasons. The climatic conditions are listed in Table 1.

**Table 1:** Climatic conditions in Shenyang

Climatic conditions	Values
Average outdoor temperature in winter	− 16.9 °C
Average temperature throughout the year	8.4 °C
Extreme maximum temperature	36 °C
Extreme minimum temperature	− 36 °C
Number of heating days	152 days
Dominant wind in winter	Northeast

Due to the COVID-19 pandemic containing measures, we only collected data of heat supply in 2017–2018 and 2018–2019 winters and presented them in Tables 2, 3. According to Tables 2, 3, we can see that there is always an imbalance between heat supply and actual need. Insufficient supply incurs dissatisfactions and overheating leads to a dramatic waste of heat and energy resources.

**Table 2:** Heat Supply Statistics in 2017–2018 Winter (10KGJ)

Month	Actual supply	Actual need	Differences	Rate (%)
11	141.04	140.25	0.79	100.56
12	168.5	155	13.5	108.71
1	165.1	165.3	− 0.2	99.88
2	138	140.3	− 2.3	98.36
3	140.7	130	10.7	108.23

**Table 3:** Heat Supply Statistics in 2018–2019 Winter (10KGJ)

Month	Actual supply	Actual need	Differences	Rate (%)
11	152.1	179.93	− 27.83	84.53
12	250.45	236.83	13.62	105.75
1	205.92	247.93	− 42.01	83.06
2	237.85	199.66	38.19	119.13
3	168.72	199.86	− 31.14	84.42

China’s adjustment on indoor temperature in winter is between 18 and 22 °C. We collected 50 indoor temperatures in a building on a winter day in 2018–2019 winter and listed them in (Table 4).

**Table 4:** 50 Indoor Temperatures of Heat Users

User	Temperature	User	Temperature	User	Temperature	User	Temperature	User	Temperature
1	21.1 °C	11	18.1 °C	21	20.3 °C	31	18.7 °C	41	16.2 °C
2	23.5 °C	12	20.2 °C	22	18 °C	32	17.3 °C	42	18.3 °C
3	21.3 °C	13	19.6 °C	23	20.7 °C	33	19.5 °C	43	18.6 °C
4	20.5 °C	14	19.1 °C	24	20.4 °C	34	15.6 °C	44	18.4 °C
5	21.6 °C	15	17.4 °C	25	15.4 °C	35	17.5 °C	45	19.7 °C
6	21.5 °C	16	20.1 °C	26	17.6 °C	36	18.5 °C	46	22 °C
7	24.7 °C	17	18.6 °C	27	19.4 °C	37	19.1 °C	47	19 °C
8	19.2 °C	18	17.9 °C	28	18.4 °C	38	17.5 °C	48	17.6 °C
9	23 °C	19	19.5 °C	29	17.2 °C	39	22.6 °C	49	15.4 °C
10	20 °C	20	17.2 °C	30	19.6 °C	40	18.1 °C	50	18.7 °C

Seeing from Table 4, we can know that 26% users are having temperatures below 18 °C resulting in an ever-growing complaint rate each year.

Here, we consider a primary network with *N* HESs and each HES serves M heat users. We optimized the inlet valve disc angles of HESs with a bi-objective hybrid algorithm based on polar bear optimization and chemical reaction optimization. As we have mentioned, adjusting valve dis angle will affect the indoor temperature and indirectly have an effect on energy consumption and online electricity volume. Therefore, a handsome save on operational cost in a CHP could be made.

**Fig. 12:**
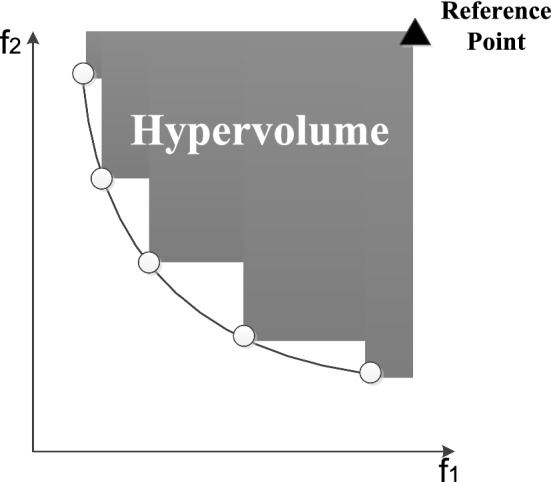
Chart of hypervolume

Hypervolume (HV) is a widely used criterion for evaluating convergence and solution distribution of a multi-objective algorithm in recent years (see Fig. 12) [49]. According to Fig. 12, we can draw a conclusion that the higher the HV, the better the Pareto frontier.

Some parameters for HA-PBO-CRO are listed in Table 5. And other parameters used in HA-PBO-CRO are set according to simple initial tests and the experiences reached in literature [24] and [29]. In HA-PBO-CRO, ${\text{PE}} = \frac{{\left( {f_{1} + f_{2} } \right)}}{2}$. Parameters of NSGA-II are set according to literature [46]. The data about the distances between heat users and their HES are shown in Appendix.

**Table 5:** Some parameters for HA-PBO-CRO

Objectives	Population size	Max iteration	HES	Runs	${\text{C}} \cdot {\text{V}}_{{{\text{max}}}}$	z	KE	Users of each HES	Crossover rate
2	100	100/200/300/500	10/20	20	45	10	5	30/60	0.6

Furthermore, we concluded ten types of satisfaction fuzzy trapezoidal functions based on investigations on 3000 local heat users (see Table 6).

**Table 6:** Satisfaction fuzzy function types

Types	Fuzzy functions
1	$${\text{ FF}}_{ij} {\text{(t}}_{i} {)}\left\{ {\begin{array}{*{20}c} {\frac{{{\text{t}}_{i} - 18}}{4}} & {{\text{t}}_{i} } & \in & {{[18,}\,{22]}} \\ 1 & {{\text{t}}_{i} } & \in & {[22,24]} \\ {\frac{{26 - t_{i} }}{2}} & {{\text{t}}_{i} } & \in & {[24,26]} \\ 0 & {{\text{t}}_{i} } & \notin & {[18,26]} \\ \end{array} } \right.$$
2	$${\text{FF}}_{ij} {\text{(t}}_{i} {)}\left\{ {\begin{array}{*{20}c} {\frac{{{\text{t}}_{i} - 18}}{2}} & {{\text{t}}_{i} } & \in & {{[18,}\,{20]}} \\ 1 & {{\text{t}}_{i} } & \in & {[20,22]} \\ {\frac{{24 - t_{i} }}{2}} & {{\text{t}}_{i} } & \in & {[22,24]} \\ 0 & {{\text{t}}_{i} } & \notin & {[18,24]} \\ \end{array} } \right.$$
3	$${\text{ FF}}_{ij} {\text{(t}}_{i} {)}\left\{ {\begin{array}{*{20}c} {\frac{{{\text{t}}_{i} - 19}}{2}} & {{\text{t}}_{i} } & \in & {{[19,}\,{21]}} \\ 1 & {{\text{t}}_{i} } & \in & {[21,23]} \\ {\frac{{25 - t_{i} }}{2}} & {{\text{t}}_{i} } & \in & {[23,25]} \\ 0 & {{\text{t}}_{i} } & \notin & {[19,25]} \\ \end{array} } \right.$$
4	$${\text{ FF}}_{ij} {\text{(t}}_{i} {)}\left\{ {\begin{array}{*{20}c} {\frac{{{\text{t}}_{i} - 17}}{2}} & {{\text{t}}_{i} } & \in & {{[17,}\,{19]}} \\ 1 & {{\text{t}}_{i} } & \in & {[19,21]} \\ {\frac{{23 - t_{i} }}{2}} & {{\text{t}}_{i} } & \in & {[21,23]} \\ 0 & {{\text{t}}_{i} } & \notin & {[17,23]} \\ \end{array} } \right.$$
5	$${\text{FF}}_{ij} {\text{(t}}_{i} {)}\left\{ {\begin{array}{*{20}c} {\frac{{{\text{t}}_{i} - 18}}{2}} & {{\text{t}}_{i} } & \in & {{[18,}\,{20]}} \\ 1 & {{\text{t}}_{i} } & \in & {[20,24]} \\ {\frac{{26 - t_{i} }}{2}} & {{\text{t}}_{i} } & \in & {[24,26]} \\ 0 & {{\text{t}}_{i} } & \notin & {[18,26]} \\ \end{array} } \right.$$
6	$${\text{FF}}_{ij} {\text{(t}}_{i} {)}\left\{ {\begin{array}{*{20}c} {\frac{{{\text{t}}_{i} - 18}}{2}} & {{\text{t}}_{i} } & \in & {{[18,}\,{20]}} \\ 1 & {{\text{t}}_{i} } & \in & {[20,22]} \\ {\frac{{26 - t_{i} }}{4}} & {{\text{t}}_{i} } & \in & {[22,26]} \\ 0 & {{\text{t}}_{i} } & \notin & {[18,26]} \\ \end{array} } \right.$$
7	$${\text{FF}}_{ij} {\text{(t}}_{i} {)}\left\{ {\begin{array}{*{20}c} {{\text{t}}_{i} } & {{\text{t}}_{i} } & \in & {[18,19]} \\ 1 & {{\text{t}}_{i} } & \in & {[19,23]} \\ {{24} - t_{i} } & {{\text{t}}_{i} } & \in & {[23,24]} \\ 0 & {{\text{t}}_{i} } & \notin & {[18,24]} \\ \end{array} } \right.$$
8	$${\text{FF}}_{ij} {\text{(t}}_{i} {)}\left\{ {\begin{array}{*{20}c} {{\text{t}}_{i} - {18}} & {{\text{t}}_{i} } & \in & {[18,19]} \\ 1 & {{\text{t}}_{i} } & \in & {[19,22]} \\ {23 - t_{i} } & {{\text{t}}_{i} } & \in & {[22,23]} \\ 0 & {{\text{t}}_{i} } & \notin & {[18,23]} \\ \end{array} } \right.$$
9	$${\text{FF}}_{ij} {\text{(t}}_{i} {)}\left\{ {\begin{array}{*{20}c} {\frac{{{\text{t}}_{i} - 18}}{2}} & {{\text{t}}_{i} } & \in & {{[18,}\,{20]}} \\ 1 & {{\text{t}}_{i} } & \in & {[20,23]} \\ {24 - t_{i} } & {{\text{t}}_{i} } & \in & {[23,24]} \\ 0 & {{\text{t}}_{i} } & \notin & {[18,24]} \\ \end{array} } \right.$$
10	$${\text{FF}}_{ij} {\text{(t}}_{i} {)}\left\{ {\begin{array}{*{20}c} {\frac{{{\text{t}}_{i} - 18}}{4}} & {{\text{t}}_{i} } & \in & {{[18,}\,{22]}} \\ 1 & {{\text{t}}_{i} } & \in & {[22,24]} \\ {25 - t_{i} } & {{\text{t}}_{i} } & \in & {[24,25]} \\ 0 & {{\text{t}}_{i} } & \notin & {[18,25]} \\ \end{array} } \right.$$

**Fig. 13:**
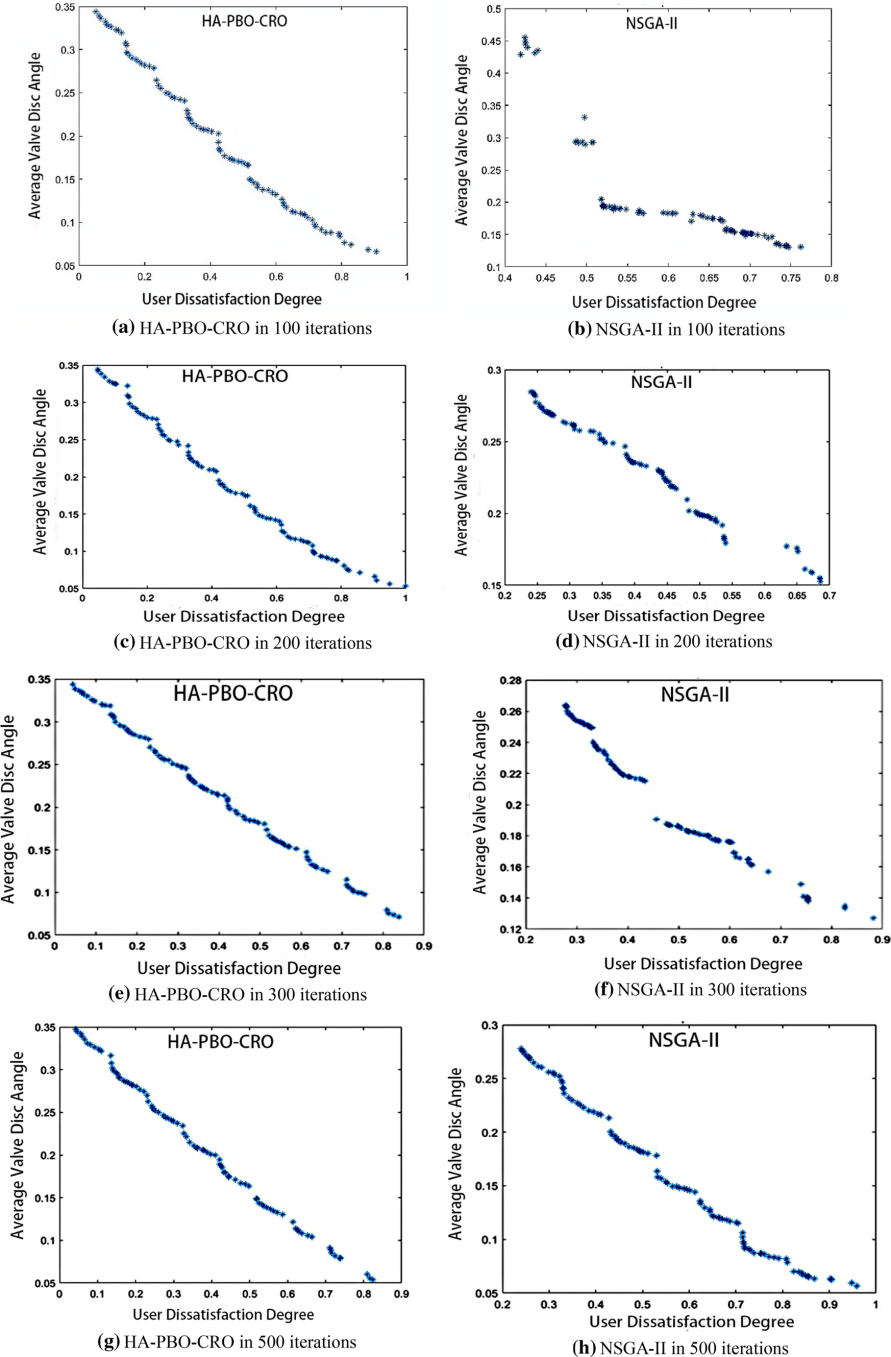
Comparative results of HA-PBO-CRO and NSGA-II. **a** HA-PBO-CRO in 100 iterations. **b** NSGA-II in 100 iterations. **c** HA-PBO-CRO in 200 iterations. **d** NSGA-II in 200 iterations. **e** HA-PBO-CRO in 300 iterations. **f** NSGA-II in 300 iterations. **g** HA-PBO-CRO in 500 iterations. **h** NSGA-II in 500 iterations

The best computational results of HA-PBO-CRO and NSGA-II after 100, 200, 300 and 500 iterations in 20 runs with 10 HESs (each has 30 users) are presented below in Fig. 13.

From the comparison of Pareto frontiers in Fig. 13, we can see that with the increase of iterations, the Pareto frontier integrity of HA-PBO-CRO is obviously better than that of NSGA-II algorithm. When the iterations are small, NSGA-II algorithm can not even give a complete Pareto frontier. Therefore, HA-PBO-CRO has an obvious advantage over NSGA-II algorithm in dealing with heat supply problem. After 20 independent runs, the average HV index is shown in Table 7.

**Table 7:** Average HV index

HES	Iterations	HA-PBO-CRO	NSGA-II
10	100	**0.531**	0.294
10	200	**0.512**	0.267
10	300	**0.553**	0.286
10	500	**0.497**	0.385

The bold values indicate that the higher the value, the better the performance

According to the comparison results of hypervolume index value (HV), it can be seen that the solution set quality produced by HA-PBO-CRO is better than that of NSGA-II algorithm.

**Fig. 14:**
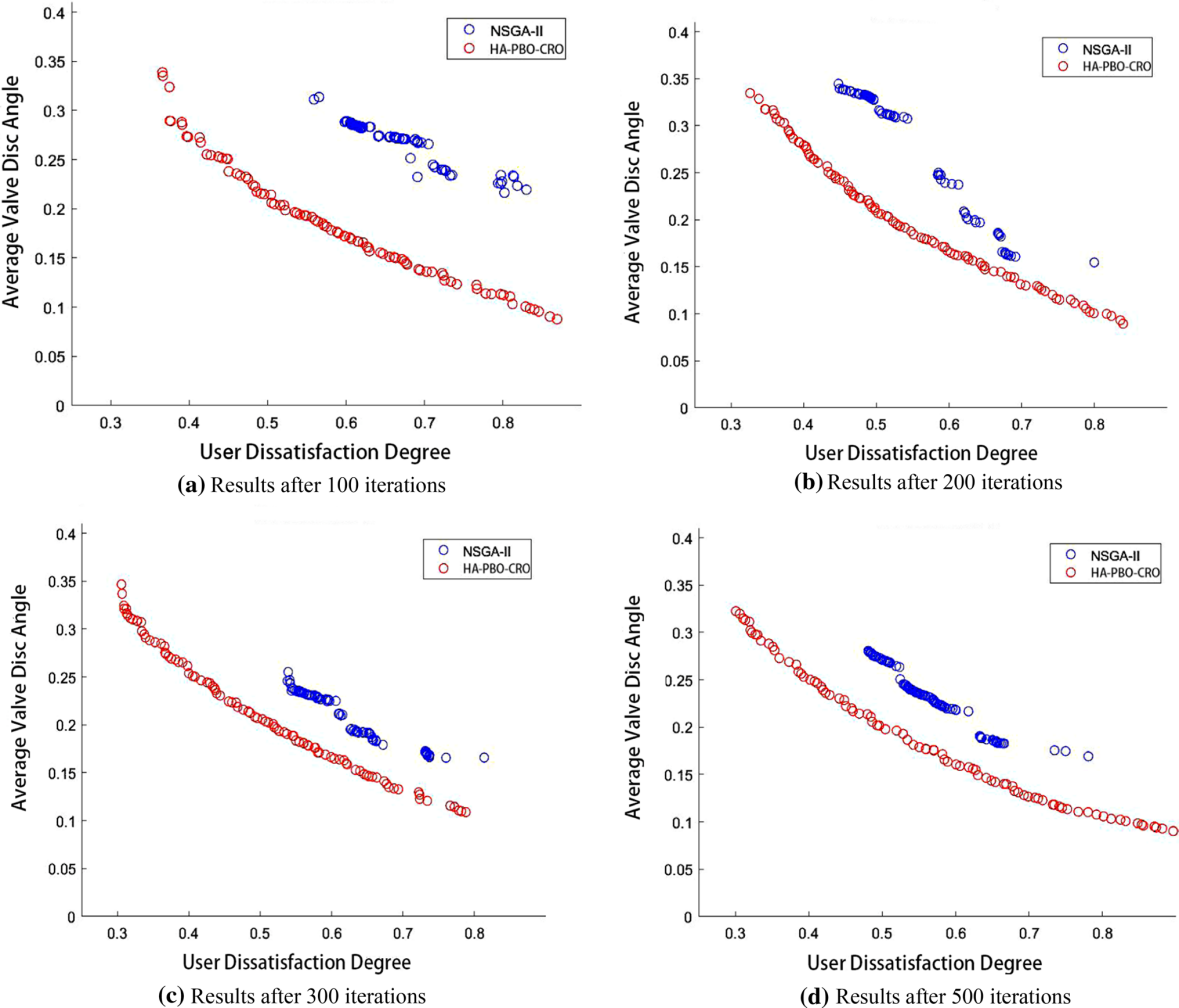
Pareto frontier comparison results of HA-PBO-CRO and NSGA-II. **a** Results after 100 iterations. **b** Results after 200 iterations. **c** Results after 300 iterations. **d** Results after 500 iterations

In order to further evaluate the performance of HA-PBO-CRO, we increase the number of HES to 20 and the number of heat users to 600 with each HES serving 60 users. The Pareto frontier comparison results are presented in Fig. 14 and the HV values are listed in Table 8.

**Table 8:** HV index values of HA-PBO-CRO and NSGA-II

HES	Iterations	HCRPBOA	NSGA-II
20	100	**0.313**	0.106
20	200	**0.334**	0.131
20	300	**0.348**	0.145
20	500	**0.398**	0.184

The bold values indicate that the higher the value, the better the performance

Two computational experiments show that HA-PBO-CRO is clearly superior to NSGA-II in solution diversity, solution distribution and convergence. And HA-POB-CRO only takes two-thirds of the computing time of NSGA-II to produce a result.

## Conclusions

In the 8-month-long cold environment, CHP plants in northern China are vital factors for local residents’ livelihoods. Through on-the-spot investigation, field research and communication with the management level of a large coal-fired CHP plant in Shenyang, we found that what is bugging them the most is the excessive electricity generated during the heat supplying process. It seems that the CHP plants should be profitable, but as a fact the fines from the government have caused serious losses in revenue. Thus, the management enquired about whether it was possible to optimize the water supplying volume with operations optimization technology to reduce the electricity entering the State Grid. Therefore, we carried out theoretical research by analyzing and examining the DHS carefully. First, we transformed the optimization on water supply volume into the optimization on inlet valve disc angle adjustment of HESs in DHS. Then, we established a bi-objective optimization model minimizing customer dissatisfaction degree and water supply cost. And we adopted a hybrid meta-heuristic algorithm to solve the mathematical model. By comparing the computational result with that of a NSGAII, we exhibited that the proposed algorithm in this paper has advantages over NSGAII in solution quality and computation efficiency. Moreover, the computational result provided significant insights for CHP management.

The research limitation in this paper is that the feasibilities of assumptions and objectives in the mathematical model need to be further verified in real-world settings. And, it is necessary to carry out more extensive and large amount of calculation tests to verify the performance and parameters of the proposed algorithm in this paper.

Future research should focus on developing online real-time heat network monitoring and operational optimization software. Therefore, our algorithm could be integrated into optimization module in the software easily as one of the optimization tools. In addition, some other interesting topics revolving CHPs are worth caring about as well. For example, research on applying Ant Lion Optimizer to work out reasonable supplier Side Bidding Strategies at Day-ahead Electricity Market. And, study on hiring whale optimization algorithm for robots’ patrolling path perception and optimization in a highly automated CHP.

## Data Availability

Yes.

## Appendix

Types of users’ satisfactory fuzzy functions and distances of users’ to 1# HES.

**Table:**

User	Distance (m)	Type
1	200	3
2	203	3
3	203	10
4	206	6
5	206	5
6	209	1
7	209	4
8	212	8
9	215	1
10	215	2
11	215	5
12	218	2
13	218	8
14	218	7
15	221	3
16	221	10
17	224	4
18	224	2
19	227	7
20	230	2
21	230	8
22	230	10
23	233	3
24	233	6
25	233	5
26	240	3
27	240	2
28	243	1
29	243	5
30	243	5

Types of users’ satisfactory fuzzy functions and distances of users’ to 2# HES.

**Table:**

User	Distance (m)	Type
1	150	3
2	150	5
3	150	9
4	153	7
5	153	5
6	153	8
7	153	4
8	156	3
9	156	1
10	156	2
11	159	5
12	159	1
13	162	4
14	162	7
15	162	3
16	165	10
17	165	4
18	165	2
19	168	3
20	168	2
21	168	10
22	171	9
23	171	5
24	174	3
25	174	6
26	160	1
27	160	2
28	163	2
29	163	1
30	163	5

Types of users’ satisfactory fuzzy functions and distances of users’ to 3# HES.

**Table:**

User	Distance (m)	Type
1	200	4
2	200	6
3	203	5
4	203	7
5	206	6
6	206	5
7	209	5
8	209	10
9	212	7
10	212	6
11	215	7
12	215	5
13	218	6
14	218	3
15	221	8
16	221	3
17	224	9
18	220	5
19	223	3
20	223	2
21	226	8
22	226	4
23	229	2
24	232	3
25	232	5
26	235	2
27	235	7
28	238	3
29	238	10
30	238	10

Types of users’ satisfactory fuzzy functions and distances of users’ to 4# HES.

**Table:**

User	Distance (m)	Type
1	300	4
2	303	6
3	303	8
4	309	5
5	309	2
6	312	1
7	312	5
8	315	3
9	318	1
10	318	7
11	400	3
12	409	4
13	409	2
14	412	9
15	412	8
16	415	4
17	418	5
18	418	7
19	421	2
20	421	1
21	449	8
22	449	3
23	452	9
24	452	5
25	455	2
26	455	4
27	458	10
28	458	6
29	461	4
30	461	3

Types of users’ satisfactory fuzzy functions and distances of users’ to 5# HES.

**Table:**

User	Distance (m)	Type
1	400	5
2	400	3
3	403	6
4	403	7
5	406	2
6	406	8
7	409	4
8	409	1
9	412	7
10	415	10
11	427	2
12	430	3
13	433	5
14	433	9
15	430	3
16	430	5
17	433	6
18	436	5
19	436	3
20	439	2
21	442	2
22	445	1
23	445	3
24	448	5
25	410	3
26	413	2
27	416	9
28	419	7
29	422	8
30	425	4

Types of users’ satisfactory fuzzy functions and distances of users’ to 6# HES.

**Table:**

User	Distance (m)	Type
1	440	7
2	443	5
3	443	1
4	446	6
5	446	7
6	449	5
7	449	4
8	452	3
9	455	10
10	458	10
11	465	8
12	468	6
13	468	10
14	471	9
15	471	7
16	474	5
17	474	4
18	477	10
19	480	2
20	480	2
21	470	2
22	470	8
23	473	9
24	476	7
25	476	4
26	479	5
27	479	6
28	482	10
29	482	5
30	485	4

Types of users’ satisfactory fuzzy functions and distances of users’ to 7# HES.

**Table:**

User	Distance (m)	Type
1	500	5
2	500	7
3	503	4
4	503	5
5	506	3
6	509	1
7	509	4
8	512	6
9	515	5
10	515	8
11	518	2
12	510	1
13	510	5
14	513	7
15	513	10
16	516	3
17	516	3
18	519	3
19	522	9
20	522	4
21	525	7
22	540	8
23	543	10
24	546	5
25	546	6
26	549	10
27	459	3
28	462	9
29	462	10
30	465	4

Types of users’ satisfactory fuzzy functions and distances of users’ to 8# HES.

**Table:**

User	Distance (m)	Type
1	550	5
2	550	7
3	553	8
4	556	9
5	559	3
6	559	10
7	562	2
8	562	6
9	565	1
10	565	5
11	568	9
12	568	3
13	571	6
14	571	7
15	574	2
16	577	3
17	580	1
18	580	8
19	583	5
20	583	2
21	590	10
22	590	8
23	593	7
24	596	3
25	596	6
26	599	1
27	602	3
28	602	5
29	605	4
30	608	6

Types of users’ satisfactory fuzzy functions and distances of users’ to 9# HES.

**Table:**

User	Distance (m)	Type
1	600	1
2	603	3
3	606	4
4	606	8
5	609	2
6	609	7
7	612	5
8	615	8
9	618	6
10	618	8
11	621	10
12	624	4
13	627	10
14	630	3
15	630	7
16	633	6
17	636	4
18	639	9
19	642	10
20	642	8
21	645	9
22	648	6
23	651	5
24	651	4
25	654	3
26	657	8
27	657	5
28	660	7
29	660	2
30	663	4

Types of Users’ satisfactory Fuzzy Functions and Distances of Users’ to 10# HES.

**Table:**

User	Distance (m)	Type
1	800	2
2	800	5
3	803	6
4	806	4
5	806	3
6	809	7
7	809	8
8	812	2
9	812	10
10	815	9
11	818	7
12	820	1
13	823	2
14	826	4
15	829	3
16	833	6
17	835	9
18	839	7
19	839	6
20	842	4
21	838	10
22	838	9
23	805	9
24	808	6
25	811	3
26	814	7
27	817	2
28	817	3
29	820	9
30	823	1

## Abbreviations

HWSVOP: Hot water supply volume optimization problem
HA-PBO-CRO: Hybrid polar bear optimization algorithm integrated with chemical reaction optimization
HES: Heat exchange station
VAAP: Valve angle adjustment problem
HWV: Hot water volume
NSGAII: Non-dominant sorting genetic algorithm
CHSs: Centralized heating systems
CHPs: Combined heat and power plants
HWCHP: Hot water from a CHP plant
MOP: Multi-objective optimization problem
PE: Potential energy
KE: Kinetic energy
EB: Energy buffer
HV: Hypervolume
